# Metabolic Screening in Psychiatric Patients: Impact of a Quality Improvement Initiative

**DOI:** 10.1192/bjo.2025.10641

**Published:** 2025-06-20

**Authors:** Ghulam Murtaza

**Affiliations:** North Dublin Mental Health Services, Dublin, Ireland

## Abstract

**Aims:** Metabolic syndrome is highly prevalent among psychiatric rehabilitation patients, with rates ranging from 40–60% globally. The condition significantly increases the risk of cardiovascular disease and type 2 diabetes, compounded by psychotropic medications, sedentary lifestyles, and poor dietary habits. Despite established guidelines from the National Institute for Health and Care Excellence (NICE), the International Diabetes Federation (IDF), and the World Health Organization (WHO), which recommend regular metabolic screening every 6 months for patients on long-term psychotropic medications, compliance with metabolic screening in psychiatric settings remains inconsistent. This audit aimed to evaluate and improve compliance with these metabolic screening practices in psychiatric rehabilitation units through targeted interventions.

**Methods:** A two-cycle audit was conducted involving 33 patients (26 males, 7 females) across five residential psychiatric units. The first cycle assessed baseline compliance with the 6-month metabolic screening guidelines, revealing significant gaps. Interventions included the implementation of a structured metabolic screening tool, GP coordination, and staff education. Screening was based on the guidelines for waist circumference, fasting glucose or HbA1c, blood pressure, triglycerides, and HDL cholesterol. The second cycle evaluated compliance with the 6-month screening interval.

**Results:** In the first cycle, only 15.15% of patients had complete metabolic screening conducted within the recommended six-month period, while 30.30% had incomplete screenings and 54.55% had missing data. Following the interventions, the second cycle showed improvements in screening compliance. In the second cycle, 66.67% of patients were screened within the recommended six-month period, while the remaining 33.33% were not screened within the recommended period of six months.

**Conclusion:** The structured metabolic screening tool and targeted interventions significantly improved compliance with metabolic screening guidelines as recommended by NICE, IDF, and the WHO. These findings emphasize the importance of regular metabolic screening and the need for continued efforts to improve adherence to established guidelines in psychiatric rehabilitation units.

